# ﻿Description and phylogenetic position of a new species, *Chrysospleniuminsularis* J.E.Jang, K.H.Lee & H.Y.Gil (Saxifragaceae), from the southern islands of South Korea

**DOI:** 10.3897/phytokeys.248.131291

**Published:** 2024-10-23

**Authors:** Ju Eun Jang, Beom Kyun Park, Kang-Hyup Lee, Hyuk-Jin Kim, Hee-Young Gil

**Affiliations:** 1 Division of Forest Biodiversity, Korea National Arboretum, Pocheon 11186, Republic of Korea Korea National Arboretum Pocheon Republic of Korea

**Keywords:** *Chrysosplenium*, morphology, new species, phylogeny, taxonomy

## Abstract

We describe a new species, *Chrysospleniuminsularis* J.E.Jang, K.H.Lee & H.Y.Gil, belonging to the family Saxifragaceae, from the southern islands of the Republic of Korea. *Chrysospleniuminsularis* is morphologically similar to *C.japonicum* (Maxim.) Makino but can be distinguished by fairly persistent bulbils, green to yellowish-green sepals, four stamens, and cylindrical papillose seeds. *Chrysospleniuminsularis* is also distinguished from *C.alternifolium* L., which is distributed in Europe, northern Russia, and the Caucasus, by the absence of stolons and green bracts. Phylogenetic analyses, based on one nuclear ribosomal (ITS) and two chloroplast (*rbc*L, *mat*K) regions, confirmed that the new species was monophyletic and that *C.insularis* and *C.alternifolium* formed a sister relationship with robust support. Herein, we provide a detailed morphological description of *C.insularis* with its corresponding geographical distribution and comparison table and figures of related species.

## ﻿Introduction

*Chrysosplenium* L. is a perennial herbaceous genus of the family Saxifragaceae, consisting of more than 70 species (Kim et al. 2019; Fu et al. 2020). Species of this genus are mainly distributed in temperate regions of the Northern Hemisphere, and their habitats are shady and humid areas in the mountains (Kim and Kim 2015; Kim et al. 2018; Zhao et al. 2022). Biogeographically, *Chrysosplenium* is known to have originated in East Asia, and several independent lineages have migrated from East Asia to the New World (Soltis et al. 2001; Deng et al. 2015; Liu et al. 2016).

The genus *Chrysosplenium* is distinguished from other genera in Saxifragaceae by its tetramerous flowers with petaloid sepals and four or eight stamens (Kim et al. 2018). However, species delimitation is often difficult in this genus because of extensive morphological variations owing to differences in growth periods and habitats (Qin et al. 2018; Kim et al. 2019; Choi et al. 2020). The genus is divided into two sections, Chrysospleniumsect.Alternifolia Franch. and C.sect.Oppositifolia Franch., based on the arrangement of the leaves (Franchet 1890). However, Hara (1957) proposed 17 series because of the high variability in the flower, capsule, and seed traits within each section. The infrageneric classification of *Chrysosplenium* species is based on several criteria, including leaf arrangement, seed surface, pedicel length, sterile branch position, capsule shape, stem surface, ovary position, stamen length, leaf surface, sepal length, and basal leaf size (Hara 1957; Fu et al. 2020). Several phylogenetic studies on Saxifragaceae genera, including *Chrysosplenium*, have been performed based on the chloroplast *mat*K region, and their results have shown that C.sect.Oppositifolia and C.sect.Alternifolia are monophyletic (Nakazawa et al. 1997; Soltis et al. 2001; Deng et al. 2015). Recently, several new species have been described based on detailed and comprehensive morphological, molecular, and cytological studies (Liu et al. 2016; Kim et al. 2018, 2019; Wakabayashi et al. 2018; Fu et al. 2020, 2021).

Thirteen *Chrysosplenium* species belonging to seven series have been recognized in the Korean Peninsula to date (Nakazawa et al. 1997; Kim et al. 2019; Choi et al. 2020; Korea National Arboretum 2021). The following are these 13 species [Chrysospleniumser.Pilosa Maxim.: *C.flaviflorum* Ohwi, *C.epigealum* J.W.Han & S.H.Kang, *C.ramosissimum* Y.I.Kim & Y.D.Kim, *C.valdepilosum* (Ohwi) S.H.Kang & J.W.Han, *C.aureobracteatum* Y.I.Kim & Y.D.Kim, *C.barbatum* Nakai; C.ser.Oppositifolia Maxim.: *C.ramosum* Maxim.; C.ser.Nepalensia Maxim.: *C.grayanum* Maxim.; C.ser.Sinica Maxim.: *C.sinicum* Maxim.; C.ser.Macrostemon H. Hara: *C.macrostemon* Maxim. ex Franch. & Sav.; C.ser.Alternifolia Maxim.: *C.japonicum* (Maxim.) Makino, *C.serreanum* Hand.-Mazz.; C.ser.Flagellifera Maxim.: *C.flagelliferum* F.Schmidt], and among them, *C.aureobracteatum*, *C.barbatum*, *C.epigealum*, *C.flaviflorum*, and *C.ramosissimum* are endemic to Korea (Chung and Kim 1988; Korea National Arboretum 2021; Chung et al. 2023).

During a floristic survey in the southern part of Korea in March 2020, we found a new *Chrysosplenium* species that is restricted to the southern islands of Korea (Jeju-do and Gageo-do Islands). This species is readily distinguished from previously known *Chrysosplenium* species in Korea by its greenish-yellow to green bracteal leaves at flowering and a cylindrical papillose seed surface. This species is most similar to *C.japonicum* (Maxim.) Makino, which belongs to the C.ser.Alternifolia, and is distributed throughout Northeast Asia, including Southeast China, Japan, Korea, Russia (Manchuria), and Taiwan (Nakazawa et al. 1997; Pan and Ohba 2001; Hsu et al. 2011). The new species, however, is clearly distinguishable from *C.japonicum* by the form of bulbils, color of sepals, number of stamens, and surface of seeds. Based on thorough literature surveys, extensive field observations, detailed analysis of floral morphology and seed coat characteristics, we designated this new species as *C.insularis* J.E.Jang, K.H.Lee & H.Y.Gil. Here, we provide a detailed morphological description and phylogenetic position of *C.insularis* and its geographical distribution.

## ﻿Materials and methods

### ﻿Material collection

Field surveys were conducted from March 2020 to March 2023. Voucher specimens were deposited at the herbarium of the Korea National Arboretum (KH, http://www.nature.go.kr/kbi/plant/smpl/KBI_2001_030100.do). Materials preserved in 70% ethanol were used to observe and measure the floral parts. Morphological observations and measurements of the new species were conducted on live and dried specimens, including the materials preserved at KH. Quantitative characteristics were measured based on at least 30 samples. The terminology used for description and comparison was referenced from Choi et al. (2020), Pan and Ohba (2001), Lozina (1939), Wakabayashi (2001), Kim et al. (2018), Kim et al. (2019), Fu et al. (2020), Fu et al. (2021).

### ﻿Microscopic observation

The seed morphology was observed under a stereomicroscope and a scanning electron microscope (SEM). The seeds were measured using a stereomicroscope (Carl Zeiss Microscopy GmbH, Stemi 508, Zeiss, Göttingen, Germany) with an Axiocam ERc 5s. Before SEM imaging, the seeds were dehydrated using 100% ethanol and sputter-coated with gold in a KIC-IA COXEM ion coater (COXEM Co., Ltd., Daejeon, Korea). SEM imaging was performed using a COXEM EM-30 PLUS+ table scanning electron microscope (COXEM) at 20 kV at the Seed Testing Laboratory of KH.

### ﻿Phylogenetic analysis

Molecular phylogenetic analyses were conducted to confirm the phylogenetic position of the new putative species of *Chrysosplenium*. Sixteen accessions of four taxa, including the new and related species, were collected from seven localities in South Korea. Total DNA was extracted from silica gel-dried leaves using the DNeasy Plant Mini Kit (Qiagen Inc., Valencia, CA) in accordance with the manufacturer’s instructions. The nrDNA region (ITS) and two cpDNA regions (*mat*K, *rbc*L) were subjected to polymerase chain reaction (PCR) (Choi et al. 2020) on a ProFlex 96-Well PCR System (Applied Biosystems, Foster City, CA, USA). The primers used and their sequences are listed in Table 1. Each reaction mixture contained AccuPower® PCR PreMix (Bioneer, Daejeon, South Korea), ca. 10 ng (1 μL) of genomic DNA, and 100 pM of primers in a total volume of 20 µL. The PCR conditions included an initial denaturation at 94 °C for 5 min, followed by 35 cycles of amplification at 94 °C for 1 min, 54 °C for 1 min, and 72 °C for 1 min, and a final extension at 72 °C for 7 min. The PCR products were visualized on 1% agarose gels and sequenced on an ABI 3730xl DNA analyzer using the ABI BigDye Terminator v3.1 Cycle Sequencing Kit (Applied Biosystems, Foster City, CA, USA). The sequences obtained were manually determined and aligned using MAFFT with Geneious Prime® 2022.1.1. (Biomatters Ltd., Auckland, NZ). The DNA sequences generated in this study have been deposited in GenBank and are indicated with an asterisk (*) in the voucher information in Table 2.

**Table 1.** Primers used for phylogenetic analysis.

Fragment	Primer	Sequence 5′ → 3′	Reference
ITS	ITS1	TCCGTAGGTGAACCTGCGG	White et al. (1990)
	ITS4	TCCTCCGCTTATTGATATGC	
*rbc*L	rbcL_1F	ATGTCACCACAAACAGAAAC	Fay et al. (1998)
	rbcL_724R	TCGCATGTACCTGCAGTAGC	
*mat*K	3F_Kim_F	CGTACAGTACTTTTGTGTTTA	K.J.Kim, pers. comm.
	1R_Kim_R	ACCCAGTCCATCTGGAAATCT	

**Table 2.** Voucher information and GenBank number of accessions used in this study (*newly generated sequences).

Taxon	Locality	Voucher information	GenBank number		
			ITS	*rbc*L	*mat*K
*C.alternifolium*	JAPAN: Shimane-ken	*DG2019032310003*	OK315466	OK315387	OK315343
*C.aureobracteatum*	KOREA: Gangwon-do, Mt. Gwangdeog	*LeeJD et al. 17127-1*	MK989508	MK989534	MK989559
	KOREA: Gangwon-do, Mt. Gwangdeog	*LeeJD et al. 17127-2*	MK989509	MK989533	MK989562
*C.barbatum*	KOREA: Jeollanam-do, Woldeung-myeon	*LeeJD et al. 17008-1*	MK989505	MK989538	MK989560
	KOREA: Gyeongsangbuk-do, Mt. Danseok	*LeeJD et al. 17020-1*	MK989506	MK989536	MK989564
	KOREA: Gangwon-do, Mt. Gwangdeog	*LeeJD et al. 17066*	MK989507	MK989537	MK989561
*C.flagelliferum*	KOREA: Gyeongsangbuk-do, Ulleung-gun, Gwanmobong	ESK21-267*	OR809214	PP133187	PP170153
	KOREA: Gyeongsangbuk-do, Ulleung-gun, Gwanmobong	ESK21-268*	OR809215	PP133188	PP170154
	KOREA: Gyeongsangbuk-do, Ulleung-gun, Seonginbong	AP22-025*	OR809213	PP133186	PP170152
	KOREA: Gyeoggi-do, Mt. Cheonma	*LeeJD et al. 17014*	MK989499	MK989530	MK989585
	KOREA: Gangwon-do, Mt. Cheongtae	*LeeJD et al. 17052-1*	MK989500	MK989529	MK989583
	KOREA: Gyeongsangbuk-do, Ulleung-do	*LeeJD et al. 17122*	MK989501	MK989531	MK989584
*C.flaviflorum*	KOREA: Gangwon-do, Mt. Pokkye	ESK21-182*	OR809216	PP133189	PP170155
	KOREA: Gangwon-do, Mt. Pokkye	ESK21-183*	OR809217	PP133190	PP170156
	KOREA: Gangwon-do, Mt. Pokkye	ESK21-184-1*	OR809218	PP133191	PP170157
	KOREA: Gangwon-do, Mt. Pokkye	ESK21-184-2*	OR809219	PP133192	PP170158
	KOREA: Chungcheongbuk-do, Mt. Gyemyeong	*LeeJD et al. 17030*	MK989513	MK989542	MK989569
	KOREA: Gyeongsangbuk-do, Mt. Cheonglyang	*LeeJD et al. 17039*	MK989514	MK989540	MK989567
	KOREA: Gangwon-do, Mt. Chiak	*LeeJD et al. 17048*	MK989515	MK989541	MK989568
*C.grayanum*	JAPAN: Hokkaido, Sapporo, Mt. Maruyama	*Nakamura 16401*	MK989524	MK989554	MK989574
	JAPAN: Hokkaido, Sapporo, Mt. Maruyama	*Nakamura 16402*	MK989523	MK989553	MK989575
	JAPAN: Hyogo prefecture, Sasayama	*Lee JH & JS Shin s. n.*	MK989525	MK989551	MK989576
	KOREA: Jeollanam-do, Mt. Cheongtae	*LeeJD et al. 17090-1*	MK989522	MK989550	MK989579
	KOREA: Jeollanam-do, Mt. Cheongtae	*LeeJD et al. 17090-2*	MK989520	MK989555	MK989578
	KOREA: Jeollanam-do, Mt. Cheongtae	*LeeJD et al. 17090-3*	MK989521	MK989552	MK989577
*C.griffithii*	CHINA	*13PXD035*	MH809138	MN185317	MN451058
*C.insularis*	KOREA: Jeju-do, Seogwipo-si, Hogeun-dong	SOK-2022-175*	OR809225	PP133198	PP170164
	KOREA: Jeju-do, Seogwipo-si, Hogeun-dong	J.E.Jang et al. 230322*	OR809226	PP133199	PP170165
	KOREA: Jeollanam-do, Gageodo	K.H.Lee 230514-1*	OR809227	PP133200	PP170166
	KOREA: Jeollanam-do, Gageodo	K.H.Lee 230514-2*	OR809228	PP133201	PP170167
*C.japonicum*	KOREA: Gyeonggi-do, Mt. Cheonma	J.E.Jang 230325-1*	OR809220	PP133193	PP170159
	KOREA: Gyeonggi-do, Mt. Cheonma	J.E.Jang 230325-2*	OR809221	PP133194	PP170160
	KOREA: Gangwon-do, Wonju-si	S.R.Lee et al. 230420-1*	OR809222	PP133195	PP170161
	KOREA: Gangwon-do, Wonju-si	S.R.Lee et al. 230420-2*	OR809223	PP133196	PP170162
	KOREA: Gangwon-do, Wonju-si	S.R.Lee et al. 230420-3*	OR809224	PP133197	PP170163
	KOREA: Jeollabuk-do, Mt. Chaegye	*LeeJD et al. 17022*	MK989502	MK989548	MK989586
	KOREA: Chungcheongnam-do, Palbong-myeon	*LeeJD et al. 17025-1*	MK989504	MK989549	MK989587
*C.kamtschaticum*	JAPAN: Hokkaido, Sapporo, Mt.Maruyama	*Nakamura 16403*	MK989516	MK989539	MK989566
*C.ramosum*	KOREA: Chungcheongbuk-do, Daegang-myeon Goseong	*LeeJD et al. 17097-1*	MK989517	MK989543	MK989571
	KOREA: Gangwon-do, Mt. Taegi	*LeeJD et al. 17147*	MK989518	MK989545	MK989573
	KOREA: Gyeongsangbuk-do, Mt. Irwol	*LeeJD et al. 17205-1*	MK989519	MK989544	MK989572
*C.sinicum*	KOREA: Jeju-do, Haean-dong	*LeeJD et al. 17043*	MK989528	MK989557	MK989582
	KOREA: Gangwon-do, Mt. Cheongtae	*LeeJD et al. 17051-1*	MK989526	MK989556	MK989580
	KOREA: Chungcheongbuk-do, Gagok-myeon	*LeeJD et al. 17086*	MK989527	MK989558	MK989581
*C.valdepilosum*	KOREA: Gangwon-do, Mt. Taegi	*LeeJD et al. 17053-1*	MK989512	MK989535	MK989563
	KOREA: Jeollabuk-do, Mt. Jiri	*LeeJD et al. 17057*	MK989510	MK989532	MK989565
*Peltoboykiniatellimoides*	JAPAN: Nagano, Kiso-Fukushima,	*Okuyama 035251*	AB248847		
	CHINA: Zhejiang, Suichang County	*XXL170002-1*		MZ779205	

We also included 32 accessions of 13 *Chrysosplenium* species deposited in GenBank and selected *Peltoboykiniatellimoides* (Maxim.) Hara as the outgroup (Soltis et al. 1996). A total of 47 accessions from 14 taxa were used for the phylogenetic analysis. Details of the voucher information and GenBank accession numbers of the species used in this study are provided in Table 2. Phylogenetic analyses were performed using the maximum likelihood (ML) method. For the ML analysis, the best-fit model was identified using ModelFinder in Phylosuite (Kalyaanamoorthy et al. 2017; Zhang et al. 2020). ML phylogenies were inferred using IQ-TREE (Nguyen et al. 2015) under the GTR+ F + R3 model in the ITS regions and the TIM+F+R2 model in the combined chloroplast regions (Minh et al. 2013).

## ﻿Results and discussion

### ﻿Taxonomic treatment

#### ﻿ Chrysosplenium insularis

Taxon classificationPlantaeSaxifragalesSaxifragaceae

J.E.Jang, K.H.Lee & H.Y.Gil sp. nov.

B5BC1FD3-D041-526D-8B66-D363DFB74D04

urn:lsid:ipni.org:names:77350706-1

Figs 1 , 4A–F

##### Diagnosis.

*Chrysospleniuminsularis* differs from *C.japonicum* in having fairly persistent bulbils, green to yellowish-green sepals, four stamens, and cylindrical papillose seeds.

##### Type.

Korea • Jeju, Seogwipo-si, Hogeun-dong; 33.25084, 126.54434; elev. 58 m; 25 Mar 2020 [fl]; *Kang-Hyup Lee JJ-200325-001* [holotype KH (Fig. 2); isotypes, 3 sheets, KH].

##### Description.

Perennial herbs, hermaphroditic, 5–15 cm tall. Bulbils present near stem base, fairly persistent, pink, turning darkish brown, pilose. Roots fibrous, white. Stems erect, cespitose, light green to green, sparsely hairy, without stolons. Basal leaves of flowering stems 1–6, opposite, simple, estipulate; petiole 3–9 cm long, glabrescent or sparsely hairy; blade reniform, 13–20 mm × 15–25 mm, apex rounded and often retuse, margins dentate to crenate, 13–17 teeth, base cordate, adaxially green, pilose, abaxially pale green, subglabrous. Cauline leaves of flowering stems 1–4, alternate, simple, estipulate; petiole 5–22 mm long, glabrescent or sparsely hairy; blade flabellate to reniform, 7–12 mm × 11–18 mm, apex retuse and often rounded or obtuse, margins dentate to crenate, 9–13 teeth, base cordate to broadly cuneate, adaxially green, pilose, abaxially pale green, subglabrous. Inflorescences terminal, 6–14 flowered cyme, surrounded by leaf-like bracts; peduncles 4.59–18.54 mm long; pedicels 0.5–1.5 mm long, sparsely pilose; bracteal leaves by inflorescence 3, petiole 0.2–4.7 mm long, glabrescent or sparsely hairy; blade subflabellate to orbicular, 2–18 × 2–14 mm, apex truncate and often retuse, margins dentate to crenate, 5–9 teeth, base broadly cuneate to subcordate, adaxially green, sparsely pilose to glabrescent, abaxially pale green, subglabrous. Flowers 4-merous, actinomorphic; sepals petaloid 4, free, erect to subspreading, ovate to broadly ovate, 1.2–2.1 × 1.5–3.1 mm, apex obtuse or rounded, yellowish green to green, glabrous; stamens 4; filaments narrow conical, 0.3–0.4 mm long; anther 0.2 mm long, yellow; pistil 2-carpellate, semi-inferior; ovary 1-locular; styles 2, free, erect, 0.2–0.3 mm long; stigma round; disc present. Capsules 2-lobed, horn shaped, lobes subequal, 2.8–3.7 × 3.8–5.2 mm long, green, glabrous, dehiscent along the adaxial suture. Seeds numerous, ovoid-ellipsoid, 0.7–0.9 × 0.5–0.6 mm, brown to dark brown, cylindrical papillose on smooth surfaces.

**Figure 1.**
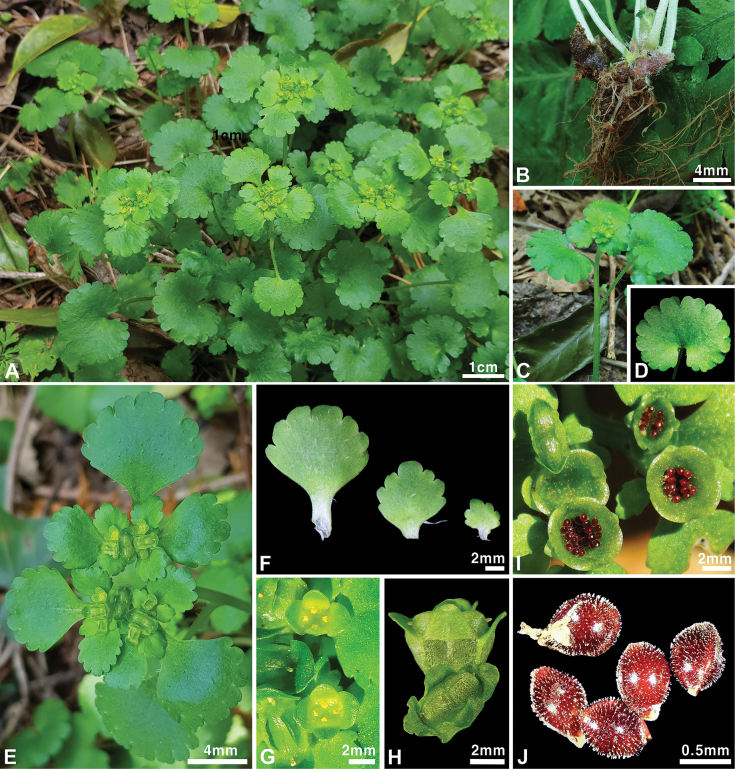
*Chrysospleniuminsularis***A** habit **B** bulbils **C** stem **D** basal leaf **E** inflorescence, **F** bracteal leaves **G** flower **H, I** capsule **J** seed. Photographs by Ju Eun Jang and Kang-Hyup Lee.

##### Phenology.

Flowering and fruiting from March to May.

**Figure 2.**
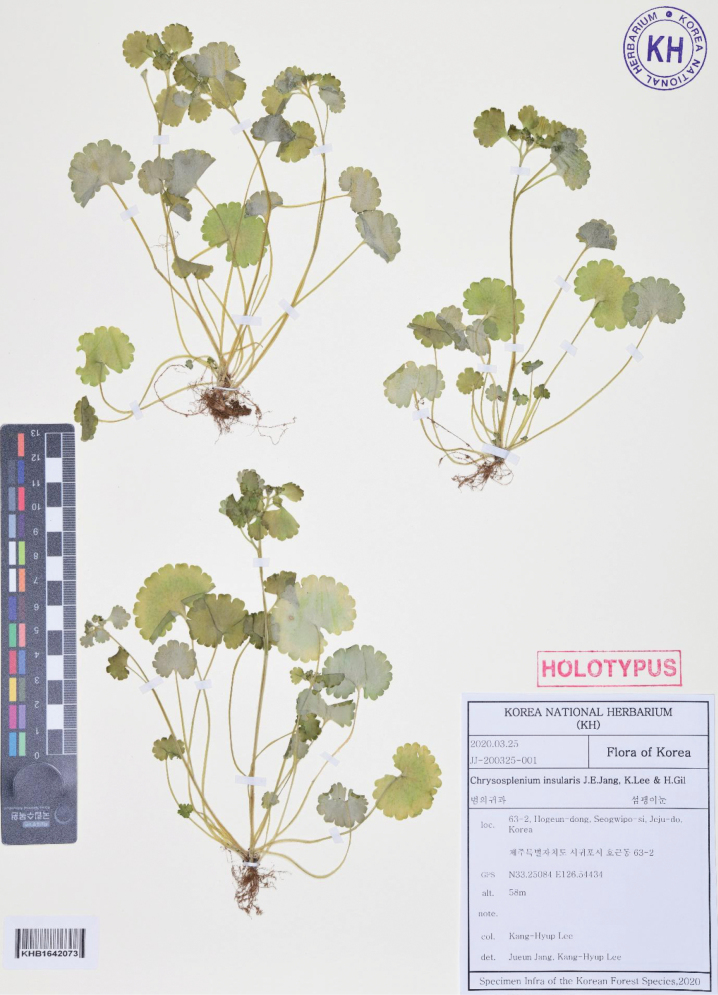
Holotype of *Chrysospleniuminsularis*.

##### Distribution and habitat.

**Figure 3.**
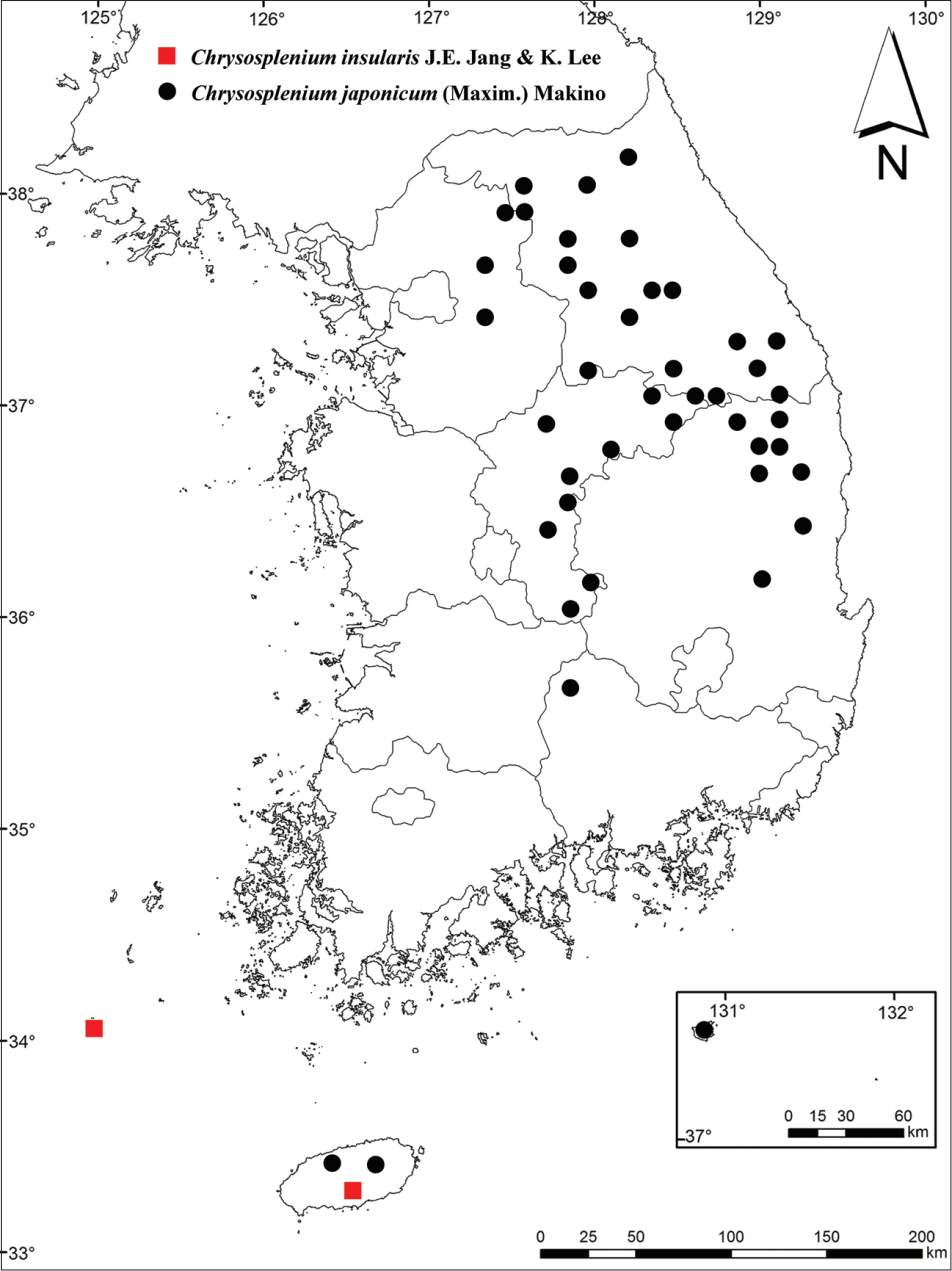
Distribution map of *Chrysospleniuminsularis* and *C.japonicum* in Korea (revised from Oh et al. 2016).

Southern coastal regions of Korea (Jeju-do and Gageo-do Islands). Forests, wet places in forests, shaded places on the riverside (Fig. 3).

##### Etymology.

The specific epithet “*insularis*” refers to its distribution on islands.

##### Vernacular name.

Island golden saxifrage: Seom-gwaeng-i-nun (섬괭이눈).

##### Morphological assessment.

**Figure 4.**
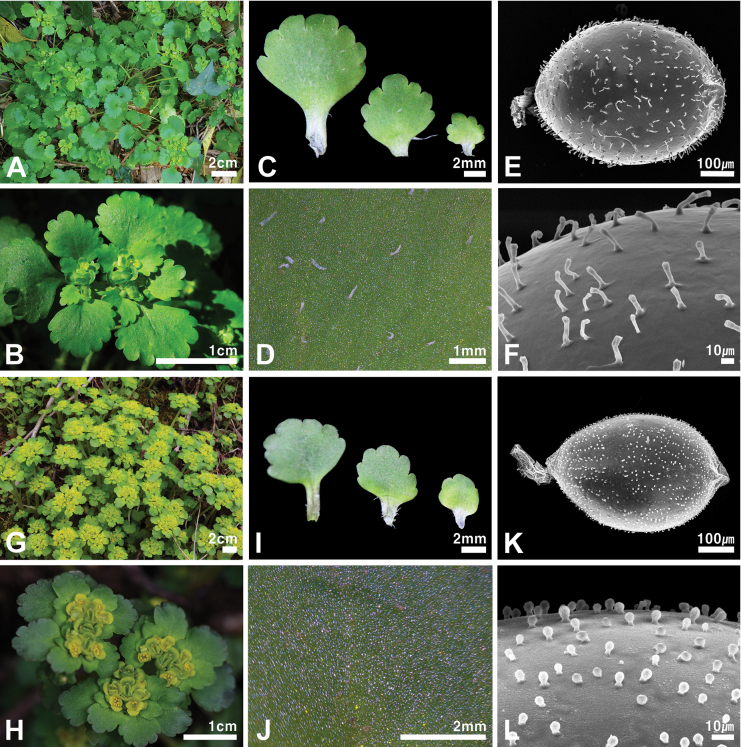
Comparative photographs of the habit (**A, G**), inflorescence (**B, H**), bracteal leaves (**C, I**), surface of bracteal leaves (**D, J**), and seed (**E, F, K, L**) of *Chrysospleniuminsularis* (**A–F**) and *C.japonicum* (**G–L**). Photographs by Ju Eun Jang and Kang-Hyup Lee.

Among the species distributed in Korea, *Chrysospleniuminsularis* is morphologically similar to *C.japonicum* in terms of leaf arrangement, leaf margin, and bracteal leaf color. Despite these similarities, it is clearly differentiated by the form of bulbils [present, fairly persistent (Fig. 1B) vs. present], surface of bracteal leaves [adaxially sparsely pilose to glabrescent, abaxially subglabrous (Fig. 4D) vs. mainly glabrous (Fig. 4J)], color of sepals [green to yellowish green vs. yellowish green to yellow], number of stamens [4 vs. usually 8], and surface of seeds [cylindrical papillose (Fig. 4E, F) vs. papillose (Fig. 4K, L)]. Additionally, this new species is morphologically similar to *C.alternifolium*, which is distributed in northern Eurasia, but is distinguished by the following characteristics: stolon (absent vs. present), color of bracts (green vs. yellow), color of sepals (green to yellowish green vs. golden yellow), number of stamens (4 vs. 8), and surface of seeds [cylindrical papillose (Fig. 4E, K) vs. smooth (Fig. 4K, L)]. A comparison of the major characteristics of the new species with those of two closely related species, *C.japonicum* and *C.alternifolium*, is shown in Table 3.

**Table 3.** Major characteristics of *Chrysospleniuminsulalis* and two closely related taxa (*: data from Lozina 1939; -: none known).

Character		*C.insulalis*	*C.japonicum*	C.alternifolia*
Bulbils		present, fairly persistent	present	-
	color	pink, turning to darkish brown	pink	-
Stolon		absent	absent	present
Bracteal leaves	color	green	yellowish green	yellow
	surfaces	adaxially sparsely pilose to glabrescent, abaxially subglabrous	mainly glabrous	mainly glabrous
Sepals	color	green to yellowish green	yellowish green to yellow	golden yellow
Stamens	number	4	usually 8	8
Seeds	surfaces	cylindrical papillose	papillose	smooth
Fl. and fr.		Mar. to May	Apr. to Jun.	Apr. to Jul.

##### Phylogenetic analysis.

**Figure 5.**
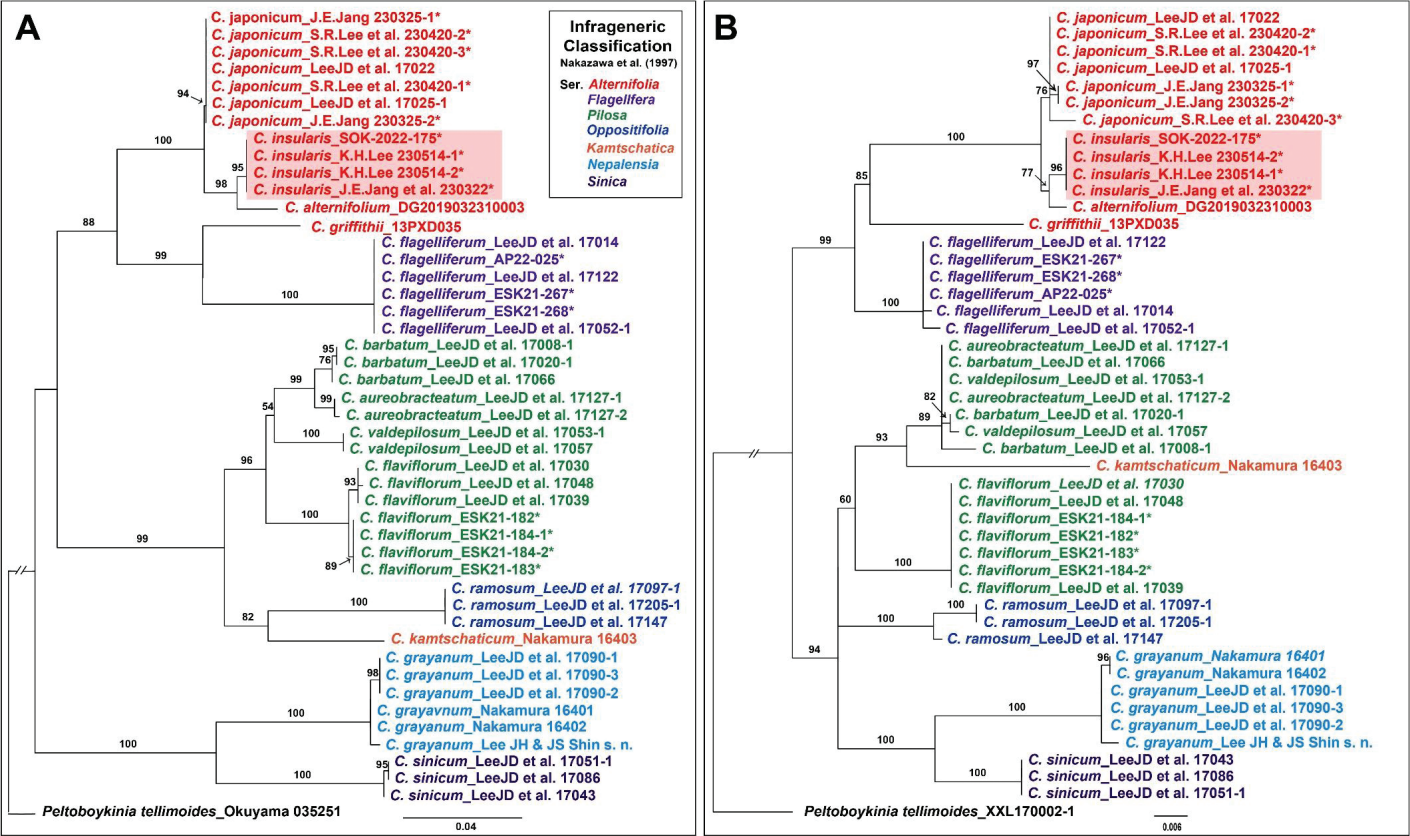
Phylogenetic tree of *Chrysospleniuminsularis* and related taxa based on ITS regions and combined CP regions (*mat*K and *rbc*L) **A** ITS region **B** combined CP regions (*mat*K and *rbc*L). The numbers above the branches are bootstrap values (BS > 50%) by the maximum likelyhood method. Newly generated sequences in this study are shown with an asterisk, and the new species are marked with a red box. The voucher information of all samples used in the analysis is indicated after the scientific names.

In total, 48 sequences of three regions (ITS, *mat*K, and *rbc*L) were newly obtained from the 16 accessions of *Chrysospleniuminsularis* and the three related taxa. We also used 93 sequences from 32 accessions obtained from GenBank (12 species of *Chrysosplenium* and one *Peltoboykiniatellimoides* as an outgroup) for the phylogenetic analysis. The aligned matrix of the ITS region and combined chloroplast regions (*mat*K and *rbc*L) contained 635 and 1407 characters, respectively. We found 242 variable sites and 193 parsimony-informative sites in the ITS regions, whereas 172 variable sites and 104 parsimony-informative sites were found in the combined chloroplast regions. The GC ratios were 46.2% and 37.4% for the ITS and combined chloroplast regions, respectively. The phylogenetic tree (Fig. 5) revealed a topology similar to that obtained in a previous study (Choi et al. 2020). The phylogenetic results showed some topological incongruence between the ITS and combined CP trees. In the ITS tree, the most basal clade (BS = 100%) included the monophyletic *C.grayanum* and *C.sinicum* and showed a sister relationship with other *Chrysosplenium* species. However, the CP tree was divided into two clades, with *C.grayanum* and *C.sinicum* sharing the most common ancestors with the C.ser.Pilosa, *C.kamtschaticum*, and *C.ramosum* (BS = 94%). The phylogenetic relationships among the three subclades were not fully resolved, and the C.ser.Pilosa. was not monophyletic, embedding *C.kamtschaticum*. Furthermore, the series *Alternifolia* was monophyletic in the CP tree but not in the ITS tree. Both trees strongly supported the monophyly of *C.insularis* (BS = 95% in ITS, BS = 96% in CP), and it shared the most common ancestor with *C.alternifolium* distributed in Japan (BS = 98% in ITS, BS = 77% in CP). The phylogenetic trees revealed that *C.insularis* formed an independent monophyletic clade from closely related taxa (i.e., *C.japonicum* and *C.alternifolium*), suggesting the newly recognized species of *Chrysosplenium* (Fig. 5).

##### Additional specimens examined.

*Chrysospleniuminsularis* (*Paratypes*): Korea • Jeonnam, Sinan-gun, Heuksan-myeon, Gageodo-ri; 14 May 2023; *K.H.Lee 230514-1* (KH). • Jeju, Seogwipo-si, Hogeun-dong; 28 Apr. 2020; *PBK0118-001* (KH). • Jeju, Seogwipo-si, Hogeun-dong; 22 Mar. 2022; *Hanon-220322-011* (KH) • Jeju, Seogwipo-si, Hogeun-dong; 22 Mar. 2023; *J.E.Jang et al. 230322-1* (KH).

*Chrysospleniumjaponicum*: Korea • Gyeonggi, Gwangju-si, Chowol-eup, Mugap-ri, Mugapsan; 24 Apr. 2007; *HNHM-A-158* • Gwangju-si, Toechon-myeon, Cheonjinam; 7 Apr. 2000; *KNAH014041* • Gwangju-si, Toechon-myeon, Usan-ri, Aengjabong; 11 Apr. 2004; *kjs040141* (KH) • Incheon-si, Ongjin-gun, Jawoldo Isl.; 8 Apr. 2009; *NAPI-2009-1214* (KH) • Incheon-si, Ongjin-gun, Deokjeok-myeon, Mungap-ri, Gitdaebong; 9 Apr. 2014; *Park140230* (KH) • Incheon-si, Ganghwa-gun, Ganghwado Isl.; 20 Apr. 2006; *LeeGH6-35* (KH) • Gyeonggi, Namyangju-si, Onam-eup, Cheonmasan; 17 Apr. 2009; *ParkSH90273* (KH) • Gyeonggi, Namyangju-si, Onam-eup, Palhyeon-ri; 25 May 2023; *J.E.Jang 230325-1* (KH) • Gyeonggi, Namyangju-si, Joan-myeon, Ungilsan; 11 Apr. 2009; *Y.M.Kang s.n.* (KH) • Gyeonggi, Gwacheon-si, Makgye-dong, Cheonggyesan; 8 Apr. 2006; *KHUS20110475* (KH) • Gangwon, Pyeongchang-gun, Yongpyeong-myeon, Jaesan-ri, Geumdangsan; 17 Apr. 2012; *JSY120434* (KH) • Jeongseon-gun, Imgye-myeon; 23 Apr. 2011; *0307013* (KH) • Gangwon, Taebaek-si, Hasami-dong, Deokhangsan; 23 Apr. 2005; *kjs050052* (KH) • Gangwon, Wonju-si, Panbu-myeon, Geumdae-ri; 20 Apr. 2023; *S.R.Lee et al. 230420-1* (KH) • Chungbuk, Danyang-gun, Danyang-eup, Suchon-ri, Sobaeksan; 17 Apr. 2005; *Sobaeksan-050417-070* (KH) • Chungbuk, Chungju-si, Sotae-myeon, Boktan-ri; 12 Apr. 2012; *Namhan-548* (KH) • Gyeongbuk, Gunui-gun, Bugye-myeon, Dongsan-ri, Palgongsan; 22 Apr. 2006; *CBU-070308* (KH) • Gyeongbuk, Bonghwa-gun, Myeongho-myeon, Bugok-ri, Cheongnyangsan; 27 Mar. 2006; *CBU-070519* (KH) • Chungbuk, Cheongsong-gun, Hyeonseo-myeon, Bohyeonsan; 22 Apr. 2006; *K.O.Yoo s.n.* (KH) • Jeonbuk, Namwon-si, Ayeong-myeon, Gusang-ri, Bonghwasan; 1 May 2007; *HNHM-A-283* (KH).

### ﻿Key to the species of *Chrysosplenium* in South Korea modified from Choi et al. (2020)

**Table:** 

1a	Cauline leaves alternate	**2**
2a	Leaves heterophyllous; sterile branches developed; caluline and bracteal leaves 2–5 lobed	***C.flagelliferum***
2b	Leaves isophyllous; sterile branch absent; cauline and bracteal leaves not lobed with 8–12 teeth	**3**
3a	Sepals green; stamens 4	***C.insularis***
3b	Sepals yellowish green or golden yellow; stamens 8	**4**
4a	Stolons present; sepals golden yellow; seed surface smooth	***C.alternifolium***
4b	Stolons absent; sepals yellowish green; seed surface papillose	***C.japonicum***
1b	Cauline leaves opposite	**5**
5a	Sepals green, spreading; capsules cup-shaped	***C.ramosum***
5b	Sepals yellow, erect; capsules horn-shaped	**6**
6a	Plants glabrous	**7**
7a	Stamens 4 (-6); cylindrical papillae with roundish head at the tip on smooth seed	***C.grayanum***
7b	Stamens 8; cylindrical papillae with truncate tip on scabrous seed surfaces	**8**
8a	Sterile branches present; plant glabrous except petiole of sterile branches; stamens shorter than the sepals	***C.sinicum***
8b	Sterile branches absent; plant glabrous; stamens longer than the sepals	***C.macrostemon***
6b	Plants pubescent	**9**
9a	Seeds without tubercules	**10**
10a	Leaves of sterile branches congested at the distal end, with white variegated veins on the upper surface	***C.flaviflorum***
10b	Leaves of sterile branches distantly arranged, with silvery dotted upper surface	***C.epigealum***
9b	Seeds with tubercules	**11**
11a	Seed tubercles arranged on inconspicuous longitudinal ridges	**12**
12a	Sterile branches highly branched, ca. 30 cm long after fruiting; leaves of sterile branches with silvery dots, upper surface glabrous; bracteal leaves yellowish-green	***C.ramosissimum***
12b	Sterile branches unbranched, less than 15 cm long after fruiting; leaves of sterile branches without silvery dots, upper surface pilose; bracteal leaves bright yellow	***C.valdepilosum***
11b	Seed tubercles arranged on prominent longitudinal ridges	**13**
13a	Leaves of sterile branches distantly arranged after fruiting; bracteal leaves golden yellow, greenish yellow at flowering	***C.aureobracteatum***
13b	Leaves of sterile branches congested at the distal end after fruiting; bracteal leaves green at flowering	***C.barbatum***

## Supplementary Material

XML Treatment for Chrysosplenium insularis

## ﻿Additional information

### Author ORCIDs

Ju Eun Jang https://orcid.org/0000-0002-6229-8477

Beom Kyun Park https://orcid.org/0000-0003-1894-3296

Kang-Hyup Lee https://orcid.org/0000-0002-7189-3235

Hyuk-Jin Kim https://orcid.org/0000-0002-3177-2914

Hee-Young Gil https://orcid.org/0000-0003-3714-0827

### Data availability

All of the data that support the findings of this study are available in the main text.
